# Phrenic nerve and esophageal injury in pulsed field ablation versus radiofrequency and cryoablation

**DOI:** 10.21542/gcsp.2025.30

**Published:** 2025-06-30

**Authors:** Sarah Shadiqa, Mohammad Iqbal, Giky Karwiky, Chaerul Achmad

**Affiliations:** Faculty of Medicine, Department of Cardiology and Vascular Medicine, Universitas Padjadjaran, Bandung, Indonesia

## Abstract

**Background:** Phrenic nerve injury (PNI) and esophageal injury are serious complications associated with atrial fibrillation (AF) ablation procedures. Pulsed field ablation (PFA), a non-thermal modality, has emerged as a promising alternative to conventional thermal ablation techniques, such as radiofrequency ablation (RFA) and cryoballoon ablation (CBA).

**Objective:** To evaluate and compare the incidence of PNI and esophageal injury following PFA, RFA, and CBA in patients undergoing AF ablation.

**Methods:** A systematic search of The Cochrane Library, MEDLINE, and EMBASE databases was conducted for studies published between 2014 and 2024. Cohort and case-control studies comparing PFA with RFA and/or CBA in relation to PNI and esophageal injury were included. Risk ratios (RRs) and 95% confidence intervals (CIs) were calculated using a random-effects model.

**Results:** Eleven studies involving 4,603 patients were included in the analysis. The incidence of PNI was 0.23% with PFA, 0.63% with RFA, and 2.68% with CBA, respectively. A meta-analysis of five studies comparing PFA and CBA showed a significantly lower risk of PNI with PFA (RR 0.13, 95% CI [0.04–0.35]; *p* < 0.0001). No esophageal injury was reported in the PFA group, compared to 2.79% in the RFA group and 1.45% in the CBA group. Pooled analysis demonstrated that PFA significantly reduced the risk of esophageal injury compared to RFA (RR 0.06, 95% CI [0.01–0.29]; *p* = 0.0005) and CBA (RR 0.07, 95% CI [0.01–0.39]; *p* = 0.002).

**Conclusion:** PFA is associated with a significantly lower risk of phrenic nerve and esophageal injury than RFA and CBA.

## Introduction

Atrial fibrillation (AF) is the most common sustained arrhythmia worldwide. This condition is a major contributor to cardiovascular-related morbidity and mortality, posing an immense global health and socioeconomic burden^1^.

In patients with symptomatic AF, rhythm control therapy, along with rate control, is recommended to improve symptoms and quality of life. One strategy to maintain sinus rhythm is catheter ablation. For patients who fail to achieve adequate control with medical therapy, catheter ablation is recommended to prevent AF recurrence^2^.

The aim of therapeutic catheter ablation for cardiac arrhythmias is to permanently deactivate arrhythmogenic tissue while preserving the unaffected parts of the heart that do not contribute to the initiation or maintenance of arrhythmias and to minimize the risk of harm to adjacent structures, such as blood vessels, the esophagus, and the phrenic nerve. Catheter ablation techniques include thermal methods such as radiofrequency ablation (RFA) and cryoballoon ablation (CBA). A novel approach is nonthermal ablation, specifically pulsed-field ablation (PFA)^3,4^.

RFA delivers a high-frequency alternating current to produce heat, whereas CBA employs extreme cold to freeze the target tissue, resulting in cell death. Both procedures can be influenced by the temperature of the surrounding blood flow, as thermal energy mechanisms rely on time-dependent conductive heating and cooling. Both techniques have been proven to be superior to antiarrhythmic drugs, yet are associated with certain risks of complications, including phrenic nerve and esophageal injury^5–7^.

In contrast to RFA and CBA, pulsed-field ablation (PFA) introduces a novel non-thermal mechanism that induces electroporation. PFA delivers ultra-rapid electrical pulses that generate a strong electric field around the catheter; at specific intensities and durations, this field causes irreversible electroporation. Cellular membranes, composed of phospholipid bilayers, serve as protective barriers that prevent the diffusion of polar molecules and maintain essential cellular functions. However, exposure to an electric field disrupts this barrier, forming nanoscale pores (nanopores) that allow ionic particles to cross the membrane, disrupting homeostasis and ultimately leading to cell destruction. PFA selectively targets myocardial tissue while sparing adjacent structures because it affects only living cells. Structural components, such as the extracellular matrix and blood vessels, which lack cell membranes, remain unaffected by the electroporation process. This level of selectivity is not observed in thermal-ablation techniques^4,8–11^.

This systematic review and meta-analysis aims to compare the safety of PFA with that of thermal ablation techniques (RFA and CBA) by evaluating the incidence of PNI and esophageal injury.

## Material and Methods

### Literature search and selection criteria

We searched the Cochrane Library*,* MEDLINE, and EMBASE databases for cohort and randomized trials written in English published between 2014 and 2024. The search terms used were (“atrial fibrillation” OR “AF”) AND (“pulsed field ablation” OR “PFA” OR “non-thermal ablation”) AND ((“radiofrequency ablation” OR “RFA”) OR (“cryoablation” OR “cryo” OR “thermal ablation” OR “conventional ablation”)) AND ((“phrenic nerve injury” OR “esophageal injury”) OR (“nerve injury” OR “esophagus injury” OR “complication” OR “adverse event” OR “side effect”)).

This systematic review and meta-analysis included studies that met the following criteria: randomized controlled trials (RCTs), cluster-randomized trials, and cohort studies that directly compared PFA with RFA and/or CBA. Studies were excluded if they did not compare different ablation procedures, were review articles, meeting abstracts, animal studies, or unpublished literature.

### Data extraction and quality assessment

The following variables were extracted and recorded: study ID, study design, sample size, type of AF treated with ablation, age of participants, intervention comparator, and incidence of PNI and esophageal injury. Inconsistencies in data extraction were resolved in a consensus meeting referring to the original articles.

We assessed the risk of bias in the studies using the Newcastle-Ottawa scale. Nine of the included studies were rated as having a low risk of bias, one as having a moderate risk, and one as having a high risk.

**Figure 1.**
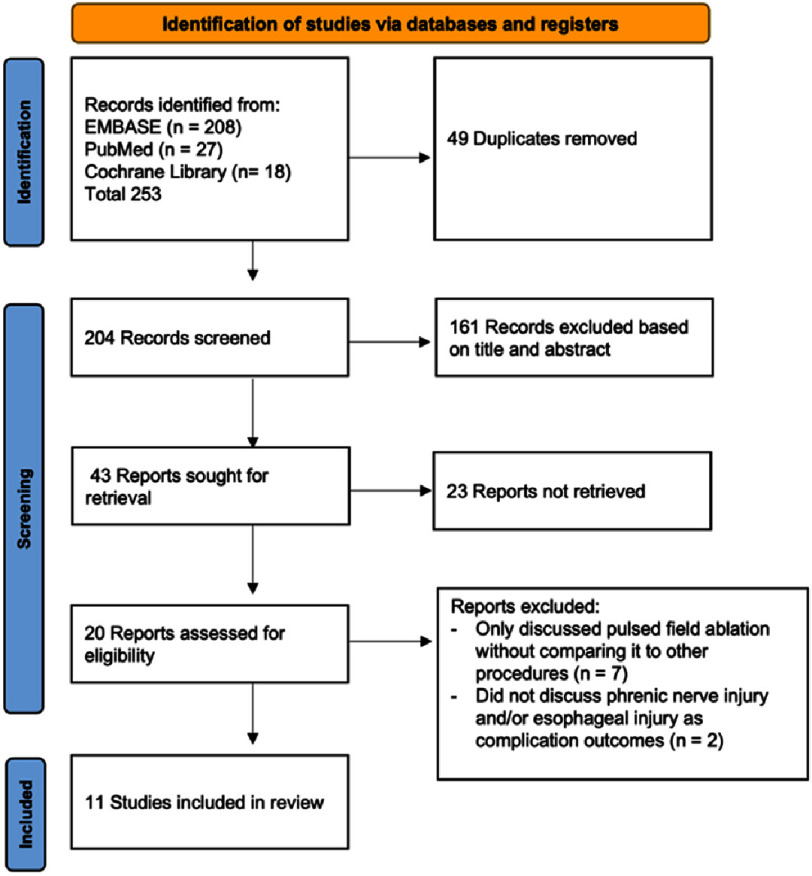
PRISMA flow diagram for the current study.

## Results

A total of 253 potentially eligible studies were identified. After removing 49 duplicates, 204 studies underwent title and abstract screening, with 184 excluded for not meeting the study objectives. Full texts of 43 studies were sought, but 23 could not be retrieved due to unavailable full texts or being conference abstracts. The remaining 20 full-text articles were assessed for eligibility. Nine studies were excluded for being non-comparative or for not reporting PNI and/or esophageal injury as a complication. Ultimately, 11 studies met the inclusion criteria and were included in the final analysis (Figure 1).

Among the 11 included studies, seven were retrospective cohort studies, three were prospective cohort studies, and one was a case-control study. The studies included in the analysis are listed in Table 1, and the extracted data are detailed in Table 2. The definitions and assessment methods of PNI and esophageal injury used in each study are summarized in Table 3 and the total incidences of PNI and esophageal injuries are summarized in Table 4.

**Table 1:** Included studies showing study design, number and sex of subjects, and AF type.

**No**	**Study ID**	**Study design**	**Subjects (n)**	**Male (n)**	**AF Type**
1	Yang et al. (2023)^12^	case control	72	49	Paroxysmal and Persistent
2	Cho et al. (2024)^13^	prospective cohort	1.237	N/A	N/A
3	Schipper et al. (2023)^14^	retrospective cohort	108	75	Paroxysmal and Persistent
4	Meininghaus et al. (2023)^15^	retrospective cohort	77	44	Paroxysmal
5	Chaumont et al. (2024)^16^	prospective cohort	301	187	Paroxysmal and Persistent
6	Reinsch et al. (2024)^17^	retrospective cohort	410	225	Paroxysmal
7	Cochet et al. (2021)^18^	prospective cohort	41	32	Paroxysmal
8	Van de Kar et al. (2023)^19^	retrospective cohort	1.714	1161	Paroxysmal and Persistent
9	Urbanek et al. (2023)^20^	retrospective cohort	400	226	Paroxysmal and Persistent
10	Maurhofer et al. (2024)^21^	retrospective cohort	200	151	Paroxysmal
11	Blockhaus et al. (2023)^22^	retrospective cohort	43	31	Paroxysmal

**Notes.**

AF atrial fibrillation N/A not available

**Table 2:** Summary of included studies.

**No**	**Study ID**	**Subjects (n)**	**Age** **(years)**	**Comparator**	**Study population (n)**			**PNI (n)**			**Esophageal injury (n)**		
					**PFA**	**RFA**	**CBA**	**PFA**	**RFA**	**CBA**	**PFA**	**RFA**	**CBA**
1	Yang et al. (2023)^12^	72	66.5 ± 14.7	RFA	36	36	N/A	0	0	N/A	0	0	N/A
2	Cho et al. (2024)^13^	1.237	N/A	RFA	492	745	N/A	2	7	N/A	0	13	N/A
3	Schipper et al. (2023)^14^	108	68 ± 12	CBA	54	N/A	54	0	N/A	2	0	N/A	0
4	Meininghaus et al. (2023)^15^	77	67 ± 10	RFA, CBA	20	24	33	0	0	1	0	12	21
5	Chaumont et al. (2024)^16^	301	62 ± 12	CBA	151	N/A	150	0	N/A	15	N/A	N/A	N/A
6	Reinsch et al. (2024)^17^	410	68 (59-74)	RFA	201	209	N/A	0	0	N/A	0	0	N/A
7	Cochet et al. (2021)^18^	41	58 ± 9	RFA, CBA	18	16	7	0	0	0	0	6	4
8	Van de Kar et al. (2023)^19^	1.714	64 (57–70)	CBA	473	N/A	1241	0	N/A	15	N/A	N/A	N/A
9	Urbanek et al. (2023)^20^	400	70 (59–77)	CBA	200	N/A	200	2	N/A	15	0	N/A	1
10	Maurhofer et al. (2024)^21^	200	62.6 ± 6.3	RFA, CBA	40	80	80	0	0	0	0	0	0
11	Blockhaus et al. (2023)^22^	43	58.1 ± 9.7	CBA	23	N/A	20	0	N/A	0	N/A	N/A	N/A

**Notes.**

RFA radiofrequency ablation CBA cryoballoon ablation PFA pulsed field ablation N/A not available

**Table 3:** Summary of outcome definitions and assessment methods.

No.	Study ID	Phrenic nerve injury	Esophageal injury
1	Yang et al. (2023)^12^	Not specified	Gastroduodenoscopy was used to assess for injury, with diagnosis based on direct endoscopic visualization for signs of perforation, bleeding, or other mucosal damage.
2	Cho et al. (2024)^13^	Not specified	Not specified
3	Schipper et al. (2023)^14^	PNI was diagnosed upon loss of phrenic nerve capture during pacing, as monitored by compound motor action potentials (CMAP).	Not specified
4	Meininghaus et al. (2023)^15^	Not specified	The diagnosis of esophageal injury was established based on the presence of pathological findings observed during endoscopic examination, as well as dysfunction in gastric electrical activity indicative of vagal nerve injury, assessed using electrogastrography (EGG).
5	Chaumont et al. (2024)^16^	PNI was diagnosed during the procedure if phrenic nerve stimulation failed to elicit normal diaphragmatic contraction, as detected by palpation and CMAP.	N/A
6	Reinsch et al. (2024)^17^	Not specified	Not specified
7	Chocet et al. (2021)^18^	PNI was assessed intra-procedurally by evaluating diaphragmatic motion using fluoroscopy. Loss of diaphragmatic motion observed on fluoroscopy categorized as PNI.	Esophageal injury was assessed using cardiac magnetic resonance (CMR) imaging with late gadolinium enhancement (LGE). Injury was defined by LGE presence on the esophageal wall and esophageal wall thickening.
8	Van de Kar et al. (2023)^19^	Manual assessment of diaphragmatic excursion during CBA procedures.	N/A
9	Urbanek et al. (2023)^20^	In PFA group PNI was noticed during spontaneous breathing, while in CBA group phrenic nerve monitored using palpation and compound motor action potential (CMAP), reduction of phrenic nerve function is when compound motor action potential decrease >30%.	Not specified
10	Maurhofer et al. (2024)^21^	Not specified	Not specified
11	Blockhaus et al. (2023)^22^	Not specified	Not specified

**Table 4:** Total incidences of phrenic nerve and esophageal injuries.

**Intervention**	**Populations**	**Phrenic nerve injuries**	**Esophageal injuries**
PFA	1.708	4 (0.23%)	0
RFA	1.110	7 (0.63%)	31 (2.79%)
CBA	1.785	48 (2.68%)	26 (1.45%)
**Total**	**4.603**	**59**	**57**

**Notes.**

RFA radiofrequency ablation CBA cryoballoon ablation PFA pulsed field ablation

A total of 4,603 subjects were included across 11 studies in this systematic review and meta-analysis, comprising patients with both paroxysmal and persistent atrial fibrillation. The average age of the participants was 64.42 years. All studies involved PFA as the intervention, compared to RFA or CBA. Three studies compared PFA with RFA, five compared PFA with CBA, and the remaining three compared PFA with both RFA and CBA.

### Phrenic nerve injury

Among the 1.708 patients who underwent PFA, four cases of PNI were reported, corresponding to an incidence of 0.23%. In the RFA group, seven out of 1.110 patients experienced PNI (0.63%), while in the CBA group, PNI occurred in 48 of 1.785 patients (2.68%).

Of the 11 included studies, only one study (Cho et al.) reported the PNI incidence for both the PFA and RFA groups; therefore, a direct meta-analysis comparison could not be performed^13^.

**Figure 2.**
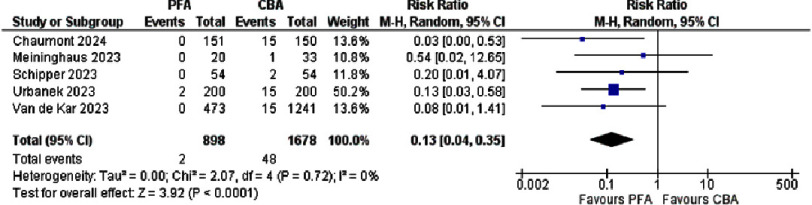
Forest plot showing phrenic nerve injury in PFA versus CBA.

A meta-analysis of five cohort studies comparing PFA and CBA for PNI (Figure 2) demonstrated a significant safety advantage of PFA. Across 898 patients treated with PFA, only two PNI were reported(0.2%), versus 48 injuries among 1.678 CBA patients (2.9%). In the study by Urbanek et al., no injuries occurred in 200 PFA patients compared with 15 of 200 CBA patients (risk ratio [RR] 0.13, 95% CI [0.03–0.58]), and Van de Kar et al. observed no PNI events in 473 PFA patients compared to 15 in 1.241 CBA patients (RR 0.08, 95% CI [0.01–1.41]). Chaumont et al. reported a similarly striking reduction (0/151 vs. 15/150; RR 0.03, 95% CI [0.00–0.53]), while smaller cohorts by Meininghaus et al. and Schipper et al. also favored PFA. When pooled using a random-effects model, the overall RR was 0.13 (95% CI [0.04–0.35], *p* < 0.0001), corresponding to an 87% relative risk reduction. Heterogeneity was negligible (I^2^ = 0%)^14–16,19,20^.

The authors of the included studies applied varying criteria to distinguish between transient and persistent PNI, utilizing different cutoff points for their classification. The following is a summary of the reported cases and their respective durations: In the CBA group, Schipper et al. documented two cases of PNI that resolved within 15 min, while Meininghaus et al. reported one case that resolved after six months. Chaumont et al. described 13 cases of PNI that resolved before hospital discharge and two cases in which dysfunction persisted beyond discharge. Van de Kar et al. reported 15 cases of PNI, all lasting more than 24 h; nine of these recovered with a mean duration of 90.7 ±24.6 days^14–16,19^.

Urbanek et al. reported two cases of PNI following PFA, both of which resolved immediately. Additionally, 15 PNI cases were reported following CBA: 12 resolved before discharge, one resolved within 18 days, another within three months, and one patient was lost to follow-up^20^.

### Esophageal injury

In the analysis of esophageal injuries following ablation procedure, a total of 57 cases were identified. PFA showed no incidence of esophageal injuries. In contrast, RFA had the highest incidence at 2.79%, whereas CBA had a lower incidence of 1.45%.

**Figure 3.**
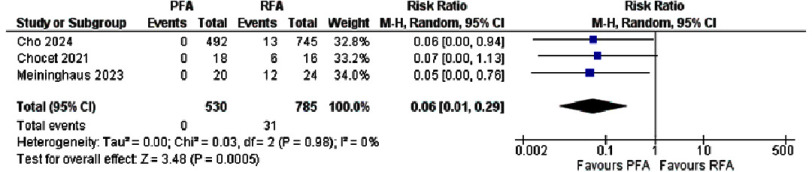
Forest plot showing esophageal injury in PFA versus RFA.

A meta-analysis of three studies evaluating esophageal injury following PFA versus RFA (Figure 3) revealed a significant safety advantage for PFA. Across 530 patients treated with PFA, no esophageal injuries were observed, compared with 31 injuries among 785 RFA recipients (0% vs. 4.0%). In the largest series, Cho et al. reported that esophageal injury occurred in 13 of 745 RFA cases versus none of 492 PFA cases (RR 0.06, 95% CI [0.00–0.94]), and similarly dramatic reductions were reported by Chocet et al. (RR 0.07, 95% CI [0.00–1.13]) and Meininghaus et al. (RR 0.05, 95% CI [0.00–0.76]).

When these studies were combined using a random-effects model, the pooled RR was 0.06 (95% CI [0.01–0.29], *p*=0.0005), indicating a 94% relative reduction in the risk of esophageal injury with PFA. There was no detectable between-study heterogeneity (I^2^ = 0%), underscoring the consistency of this finding^13,15,18^.

**Figure 4.**
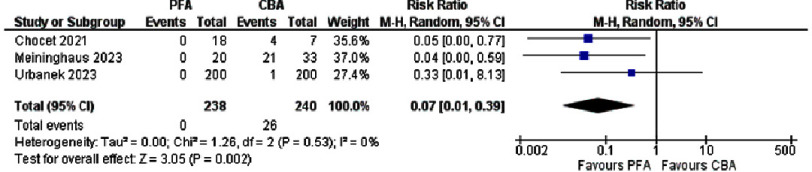
Forest plot showing esophageal Injury in PFA versus CBA.

A pooled analysis of three studies comparing PFA and CBA demonstrated a substantial reduction in esophageal injury with PFA (Figure 4). Among 238 patients treated with PFA, no esophageal injuries were reported, whereas 26 injuries occurred among 240 CBA patients (0% vs. 10.8%). In Chocet et al., esophageal injury was seen in 4 of 7 CBA cases versus none of 18 PFA cases (RR 0.05, 95% CI [0.00–0.77]). Meininghaus et al. similarly observed 21 injuries in 33 CBA patients versus none in 20 PFA patients (RR 0.04, 95% CI [0.00–0.59]). Urbanek et al. reported one case among 200 CBA patients and none among 200 PFA cases (RR 0.33, 95% CI [0.01–8.13]). When these studies were combined under a random-effects model, the overall RR was 0.07(95% CI [0.01–0.39]; *p*=0.002), corresponding to a 93% relative risk reduction with PFA. Heterogeneity was negligible (I^2^ = 0%), underscoring the consistency of PFA’s protective effect on the esophagus across diverse settings^15,18,20^.

In a study by Meininghaus et al., periesophageal edema was observed in four out of 20 patients in the PFA group, as detected by endoscopic ultrasound. However, this finding was not categorized or reported as an esophageal injury, as endoscopic examinations showed normal mucosal findings. The study also reported that within 48 h after the procedure, symptoms of gastric hypomotility, such as dysphagia, postprandial fullness, nausea, and vomiting, were observed in 28 patients (12 in the CBA group, 11 in the RFA group, and 5 in the PFA group). At 4 to 6 weeks post-procedure, symptoms resolved in 26 of the 28 patients. The remaining two patients required hospitalization due to nausea, vomiting, and epigastric pain^15^.

In the study by Cochet et al., patients underwent cardiac magnetic resonance imaging at 3-month follow-up. The results demonstrated that all previously identified esophageal lesions in the RFA and CBA groups had completely resolved^18^.

## Discussion

### Phrenic nerve injury

PNI during ablation procedures primarily occurs because of the close anatomical proximity of the phrenic nerve to the cardiac structures targeted during AF ablation, such as the superior vena cava (SVC) and right superior pulmonary vein (RSPV)^23^.

From the included studies, PNI was significantly less frequent with PFA than with CBA and RFA. The overall incidence of PNI was 0.23% with PFA, 0.63% with RFA, and 68% with CBA. A meta-analysis of five cohort studies comparing PFA and CBA further confirmed this finding, showing an 87% relative risk reduction of PNI with PFA (RR 0.13, 95% CI [0.04–0.35], *p* <0.0001), with no heterogeneity between studies (I^2^ = 0%). These results reinforce the safety advantage of PFA, particularly in minimizing PNI risk, compared with thermal ablation techniques.

In CBA, direct cold-induced injury can result from deep balloon placement near the RSPV, which can lead to nerve damage. CBA has a higher incidence of PNI than other ablation techniques. Reducing the duration of the CBA procedure can lower the risk of PNI^24,23^. In RFA, thermal injury occurs because of heat conduction to nearby nerve tissues. Compared to CBA, RFA has a lower risk of PNI; however, it is associated with a higher risk of other cardiovascular complications, such as pericardial effusion and cardiac tamponade^23^.

The selectivity of PFA is attributed to the significantly lower electroporation threshold of cardiomyocytes compared to that of other tissue types^1,1^. Although PFA selectively targets the myocardial tissue through electroporation, rare cases of PNI have been reported. This is likely caused by mechanical trauma from catheter manipulation or electrical overstimulation (hyperpolarization) of the nerve rather than thermal injury^23^. This observation helps clarify that PFA, which is widely regarded as having a high safety profile, still presents incidences of PNI, despite being at the lowest rate. In the MANIFEST-PF registry, which included 1,568 patients who underwent PFA, 0.4% experienced transient phrenic nerve injury, and 0.06% developed persistent phrenic nerve injury^25^.

PNI following ablation procedures can be either transient or persistent. Transient PNI is defined as phrenic nerve dysfunction that fully resolves before the end of the ablation procedure, whereas persistent PNI is defined as dysfunction that remains present after a 30-minute observation period following the procedure^23,26^.

With regard to the classification of PNI as either transient or persistent, Urbanek et al. reported two transient PNI cases in patients who underwent PFA, both of which resolved immediately after surgery. Schipper et al. reported two transient PNI cases following CBA that resolved within 15 min. The remaining 46 PNI cases in the CBA group persisted for >30 min after the procedure. The duration of the other PNI cases—two in the PFA group and seven in the RFA group—was not specified, thus it could not be determined whether they were transient or persistent^14–16,19,20^.

The variation in PNI type observed across different ablation modalities may be attributed to their distinct mechanisms of injury. Pansera et al. reported three cases of PNI following PFA that resolved within less than one minute. The authors proposed that this transient PNI may result from neuronal hyperpolarization, which temporarily inhibits the initiation of action potentials; however, further studies are required to confirm this hypothesis. In contrast, thermal ablation induces nerve dysfunction through dose-dependent nerve damage, which is influenced by both temperature and application duration. In RFA, permanent PNI is associated with features of acute thermal injury, such as edema, coagulation, and irreversible chromatin and cytoplasmic content damage, whereas transient PNI shows no structural evidence of nerve damage. Meanwhile, prolonged cold exposure during CBA may result in axonal loss due to Wallerian degeneration^27–29^.

### Esophageal injury

Esophageal injury during AF ablation primarily occurs because of the close spatial relationship between the esophagus and the posterior wall of the left atrium (LA), where ablation is typically performed. Although the exact mechanism of injury remains unclear, several potential causes have been identified. These include direct thermal injury, especially from RFA and CBA, ischemic injury from occlusion of small esophageal arteries, acid reflux-induced damage, mechanical trauma from catheter manipulation, and vagal nerve injury, which can impair esophageal motility^30^.

In the study by Meininghaus et al., four patients in the PFA group experienced periesophageal edema, and five presented with symptoms of gastric hypomotility 48 h following the procedure. However, these findings were not categorized as esophageal injury, as endoscopic examinations revealed normal mucosal findings with no evidence of ulceration or erosion^15^.

The most severe form of esophageal injury is atrioesophageal fistula (AEF), a life-threatening condition that typically begins with esophageal ulceration, which may develop within hours to days after the procedure^30^.

The POTTER-AF study, which involved a large population undergoing thermal ablation procedures (RFA and CBA), found that esophageal fistulas occurred more frequently after RFA than after CBA^31^.

## Limitations

A potential limitation of this review is the variability in operator experience and institutional protocols, which may have influenced the reported complication rates of the procedures. The adoption of PFA, which is relatively recent, may have involved operators who were still within the early stages of their learning curves, whereas RFA and CBA are more established techniques. Differences in procedural approaches, mapping systems, and complication monitoring across centers were not consistently reported, introducing possible unmeasured confounding factors.

In addition, most of the included studies reported only short-term outcomes, limiting the ability to assess delayed or long-term complications of the procedure. Further prospective studies with long-term follow-up are required to validate these findings.

## Conclusion

This systematic review and meta-analysis of 11 studies involving 4,603 patients demonstrates that PFA is associated with a significantly lower incidence of PNI and no reported cases of esophageal injury compared to thermal ablation techniques, RFA, and CBA. Meta-analyses revealed a statistically significant reduction in the risk of PNI with PFA compared to CBA, and esophageal injury compared to both RFA and CBA, all with negligible heterogeneity. These findings support the favorable safety profile of PFA in reducing phrenic nerve and esophageal injury during atrial fibrillation ablation. However, further prospective studies are warranted to confirm the proposed mechanisms and to evaluate the long-term outcomes of these complications.

### Author contributions

**Conceptualization:** Sarah Shadiqa and Chaerul Achmad.

**Writing - Original Draft Preparation:** Sarah Shadiqa.

**Writing - Review & Editing:** Mohammad Iqbal, Giky Karwiky, and Chaerul Achmad.

**Supervision:** Chaerul Achmad.
